# New record of *Epistylishentscheli* (Ciliophora, Peritrichia) as an epibiont of Procambarus (Austrocambarus) sp. (Crustacea, Decapoda) in Chiapas, Mexico

**DOI:** 10.3897/zookeys.782.26417

**Published:** 2018-08-16

**Authors:** Mireya Ramírez-Ballesteros, Gregorio Fernandez-Leborans, Rosaura Mayén-Estrada

**Affiliations:** 1 Laboratorio de Protozoología, Departamento de Biología Comparada, Facultad de Ciencias, Universidad Nacional Autónoma de México, Av. Universidad 3000, Circuito Exterior S/N. Coyoacán, 04510. Ciudad de México, México Universidad Nacional Autónoma de México México Mexico; 2 Posgrado en Ciencias Biológicas, Facultad de Ciencias, Universidad Nacional Autónoma de México Universidad Complutense Madrid Spain; 3 Departamento de Zoología, Facultad de Biología, Universidad Complutense, Calle José Antonio Novais 12, 28040. Madrid, España Universidad Nacional Autónoma de México Mexico Mexico

**Keywords:** ciliate, colonies, epibiosis, epistylid, Montebello

## Abstract

Epibiosis is very common between crustaceans and ciliates where the calcified surface of the crustacean body provides a suitable substrate for ciliate colonization. The aim of this contribution is to provide data about a new record between the epistylid ciliate *Epistylishentscheli* Kahl, 1935, and the crayfish Procambarus (Austrocambarus) sp. The distribution of the epistylid on the basibiont body and its cellular/colonial characteristics were analyzed. Procambarus (Austrocambarus) sp. harbored colonies of *E.hentscheli* only on the pereiopods. This is the first record of this peritrich ciliate as an epibiont on Crustacea, having been previously found on algae and fish.

## Introduction

Epibiosis is a facultative and interspecific association between two organisms, the epibiont and the basibiont, the latter providing a substrate for the attachment of the former (Wahl 2008). The basibionts are usually significantly larger than epibionts, have body surfaces that are physiologically inactive, and are sessile or slow-moving (Threlkeld et al. 1993; Wahl and Mark 1999). Epibiosis is a continuous and dynamic process in which the benefits and costs for basibionts and epibionts can change depending on environmental conditions (Fernandez-Leborans 2010).

Epibiotic associations between crustaceans and ciliates are very common, since the calcified surface of the crustacean functions as a semi-permanent substrate, providing an optimal habitat for epibionts ciliates, especially in those areas where other substrates are not suitable for long-term colonization (Fernandez-Leborans 2010). Among the ciliate epibionts of crustaceans, the Peritrichia (Fernandez-Leborans and Tato-Porto 2000a), Suctoria (Batisse 1994; Fernandez-Leborans and Tato-Porto 2000b) and Chonotricha (Fernandez-Leborans 2010) are the most commonly reported. Regarding the 13 species of the sessilid peritrich genus *Epistylis* (Table 1), so far there have been no reports of *E.hentscheli* as epibiont of crustaceans.

Ciliates of the genus *Epistylis* include colonial organisms with a non-contractile and branched stalk; each zooid has a well-defined peristomial lip and epistomial disc in the oral region, being the zooids elongated and generally in the shape of a vase (Lynn 2008). Procambarus (Austrocambarus) sp., a member of the family Cambaridae, is a freshwater decapod inhabiting dams, streams, and rivers. Species of this genus are considered important macro- invertebrates in temperate and tropical areas, participating in maintaining the balance in the food chain through the processes of degradation of the organic matter of the systems (Álvarez et al. 2012; Yazicioglu et al. 2016). The crayfish can represent up to 85% of the zoobenthic biomass, are considered strong engineers of the ecosystems, and can be considered as ecological regulators (Veselý et al. 2015).

The goal of this contribution is to provide data of *E.hentscheli* and its distribution on the body of the crayfish Procambarus (Austrocambarus) sp., including some cellular/colonial characteristics of the epistylid.

**Table 1.** Species of the genus *Epistylis* reported previously as epibionts of freshwater decapods.

Decapod host	Ciliate species	Infected body regions	Sources
***Pontastacusleptodactylus* Eschscholtz, 1823**	*Epistylis* sp.	Antennae Carapace Pleopods Telson Uropods Gills	Zaikov et al. (2000), Harlioglu (1999), Hüseyin and Selcuk (2005), Nekuie et al. (2015)
	*E.niagarae* Kellicott, 1883		
	*E.chrysemidis* Bishop & Jahn, 1941		
	*E.astaci* Nenninger, 1948		
	*E.cambari* Kellicott, 1885		
	*E.crassicollis* Stein, 1867		
***Astacusastacus* Linnaeus, 1758**	* E. astaci *	Rostrum Antennules Antennae	Fernandez-Leborans and Tato-Porto (2000a)
	*E.bimarginata* Nenninger, 1948		
	* E. crassicollis *		
***Cheraxtenuimanus* Smith, 1912**	*Epistylis* sp.	Pereiopods	Villarreal and Hutchings (1986)
***Cambarelluspatzcuarensis* Villalobos, 1943**	* E. bimarginata *	Uropod Antennules Rostrum Gill Pereiopods Pereiopod Uropod Telson	Mayén-Estrada and Aladro-Lubel (2001)
	*E.branchiophila* Perty-Stein, 1859		
	*E.carinogammari* Stiller, 1949		
	*E.gammari* Precht,1935		
	*E.lacustris* Imhoff, 1884		
	* E. niagarae *		
	*E.stammeri* Nenninger, 1948		
	*E.variabilis* Stiller, 1953		

## Materials and methods

*Sampling*. Specimen of Procambarus (Austrocambarus) sp. were collected in an artificial pond of Montebello Chiapas, Mexico [16°04.40N, 91°37.40W (DDM)], 1,507 m above sea level, during the rainy and dry seasons in years 2014–2015, being the mud and clay the principal substrate. Collections during the rainy season and the dry season were performed every three months, and in each sampling the following physical and chemical parameters were measured: water temperature, conductivity, and pH by a YSI model 85 multiparameter sonde and dissolved oxygen concentration was measured with an oximeter YSI model 55/12.

*Techniqueprocedures*. Crustaceans were transported alive to the Protozoology laboratory (Faculty of Sciences, Universidad Nacional Autónoma de México, Mexico City), and maintained alive in aquaria. Specimens were later dissected to separate the telson, pleopods, pereiopods, carapace, chelipeds, antennae, eyes, gills, and mouthparts. Peritrichs were observed with a Nikon stereoscopic microscope (SMZ 800). Photomicrographs and morphometric records were obtained using a Nikon digital camera (Digital Sight DS2Mv) adapted to a Nikon microscope (Labophot2/AX70).

Ciliates were fixed in 70% alcohol, to reveal their cellular structure with the pyridine silver carbonate technique (Fernandez-Leborans and Castro 1986), and the protargol impregnation technique (Foissner 2014). Peritrichs measurements were obtained from live and stained individuals and included: length and width of the zooid, macronucleus, stalk and also width of the peristomial collar. *Epistylishentscheli* was identified based on morphological characteristics described by Foissner et al. (1992), including the measurements of length and width of the zooids, and the width of the peristomial collar. Main morphological features of this species include the shape of the zooids and tall of the entire colony.

## Results

The physical and chemical parameters data recorded during the dry (DS) and rainy season (RS) of the pond, which were measured each three months were: temperature (DS: 18.7 °C ± 2.1, RS: 21.2 °C ± 1.8), pH (DS: 7 ± 0.5, RS 6 ± 0.4) conductivity (DS: 321 µS ± 56, RS: 243 µS ± 64), and dissolved oxygen (DS: 7.46 mg/L ± 0.9, RS:8.85 mg/L ± 0.87).

**Figure 1.**
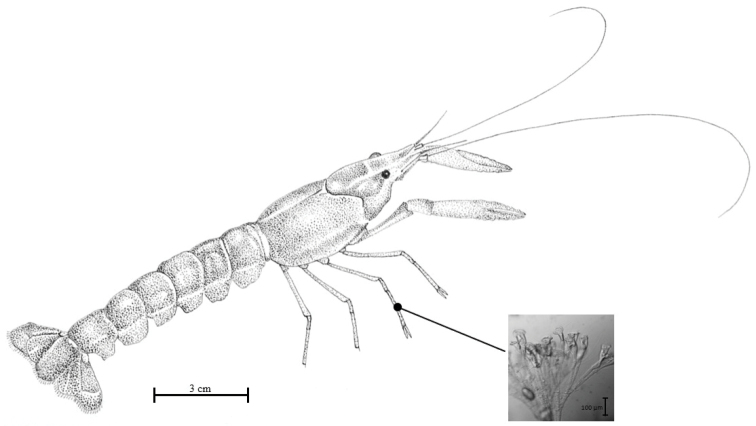
Procambarus (Austrocambarus) sp. from Montebello, Chiapas, Mexico. Dorsal view. Colonies of *Epistylishentscheli* are shown.

Ninety-six crayfish specimens were collected, 46 in the dry season and 50 in the rainy season. *Epistylishentscheli* was recorded only during the dry season of year 2015 on 36 individuals of the crayfish (prevalence of 78%), and only on pereiopods, between the merus and the carpus (Figure 1); the number of colonies on individual crayfish varied between one and three.

**Figure 2.**
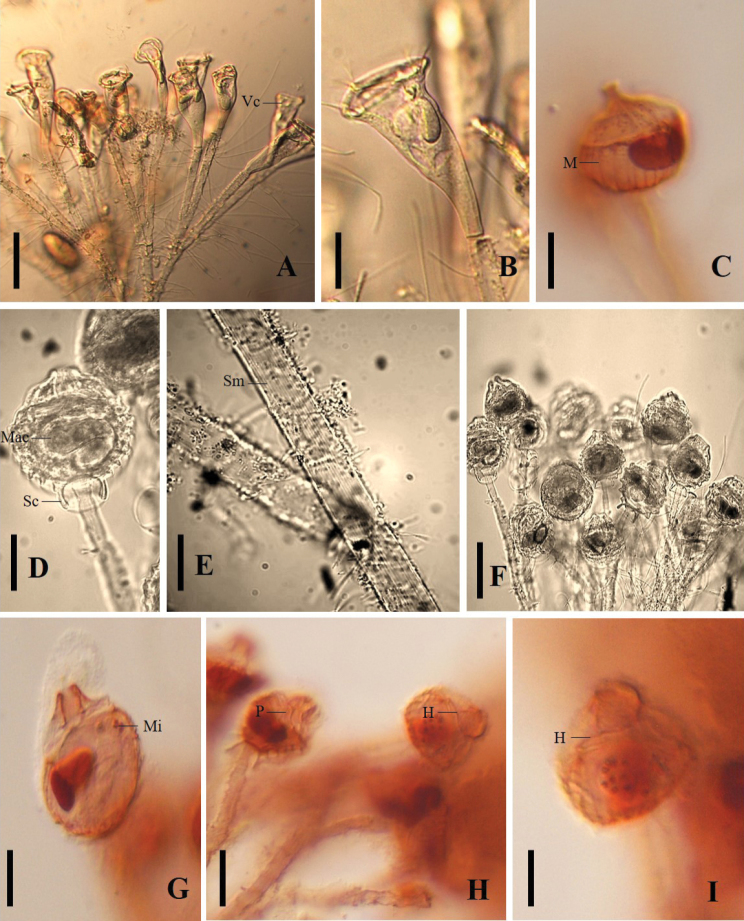
**A–B***Epistylishentscheli* in vivo, **A** colony **B** detail of zooid **C–F** zooid after silver carbonate staining **C** details of myonemes and macronucleus **D** details of stalk **E** detailed longitudinal fibers in the stalk **F** colony showing contracted zooids **G–I** protargol-stained zooids. Abbreviations: Cv. contractile vacuole; H. haplokinety; M. myonemes; Mac. macronucleus; Mi. micronucleus; Po. polykinety; Sc. scopula; Sm. stretch marks. Scale bars: 100 µm (**A**), 25 µm (**B–D, F–H**), and 10 µm (**E, I**).

Forty colonies of *E.hentscheli* with 20–30 zooids were observed with a dichotomously branched pattern, with a long and rigid main stalk that contained peripheral fibers arranged longitudinally (Figure 2E). The observation of 38 zooids in vivo showed uncontracted and trumpet-shaped zooids (Figure 2A, B), with a peristomial disc slightly raised above the peristome; and with the infundibulum reached more than half the length of the zooid (Figure 2B). The single contractile vacuole was located above the C-shaped macronucleus (Figure 2B, D).

From stained zooids we observed one spherical micronucleus located close to the central macronucleus (Figure 2G). The oral infraciliature comprised the haplokinety and polykineties running parallely, which made approximately one and a quarter turns around the peristomial disc. At the opening of the infundibulum the haplokinety separated from the polykinety (Figure 2H–I). Biometric data of *E.hentscheli* are shown in Table 2.

**Table 2.** Biometric features of *Epistylishentscheli* in vivo and after protargol staining, colonizing Procambarus (Austrocambarus) sp. (measurements in µm, n=38; Min. minimum; Max. maximum; S.D. standard deviation; C.V. coefficient of variation).

Attribute	Measurements in vivo					Measurements after protargol staining				
	Min.	Max.	Mean	S.D.	C.V	Min.	Max.	Mean	S.D.	C.V
**Zooid length**	111	140	120	21.4	0.17	32	76	54	14.6	0.27
**Zooid width**	58	87	73	9.85	0.13	65	91	78	7.43	0.09
**Width of peristomial collar**	62	90	76	9.71	0.12	20	34	28	4.06	0.14
**Macronucleus width**	4	6	5	0.88	0.17	3	5	4	0.81	0.20
**Macronucleus length**	37	44	40	2.46	0.06	25	34	30	3.05	0.10
**Primary stalk length of colony**	511	700	606	62.7	0.10	511	700	606	62.7	0.10
**Primary stalk width of colony**	7.3	7.5	7.4	0.08	0.01	7.3	7.5	7.4	0.08	0.01

## Discussion

The current study represents the first ever record of *Epistylishentscheli* as an epibiont of Crustacea. Some ciliate species have been recorded on decapods in Mexico (López-Ochoterena and Ochoa-Gasca 1971; Mayén-Estrada and Aladro-Lubel 1998, 2000, 2001, 2002, Vidal-Martínez et al. 2002), but there are no records from Chiapas state. *Epistylishentscheli* has been previously recorded as an ectoparasite of *Cyprinuscarpio*(Chordata, Cyprinidae) in Mexico (Herróz-Zamorano 1998), and Zaleski and Claps (2001) found this species on *Enteromorpha* sp. (Plantae, Chlorophyta) in Argentina.

*Epistylishentscheli* colonies were formed by 20–30 zooids each and were attached to the pereiopods of Procambarus (Austrocambarus) sp. It is likely that the constant movement of these pereiopods provide a constant water flow carrying suspended food particles and oxygen to the ciliates. In contrast, the dorsal surface of the basibiont is subject to comparatively little water flow and also is exposed to more abrasion forces, possibly preventing the ciliate attachment. The ciliate colonies of *E.hentscheli* on the pereiopods were very long, with a stalk of 600 μm. This result agrees with that of Camacho and Chinchilla (1990) who reported that the location of epibiont ciliates is determined by the structural characteristics of the ciliates, and genera such as *Zoothamnium*, *Vorticella*, and *Epistylis*, with long stalks, adhere to body regions exposed to water currents, such as uropods and pereiopods. Fernandez-Leborans and Gabilondo (2006) and Key et al. (1997) also indicated that the adhesion site of the ciliates not only depends on the epibionts, but also depends on a series of other characteristics such as the locomotion, shape, molting period, sex, and the behavior of the crustacean.

Epibiosis is a facultative association, in which both participants gain advantages but also have disadvantages from this interaction (Fernandez-Leborans 2010). In this case, the advantage for the epibiont *E.hentscheli* is some protection against predators and a constant water flow providing food and oxygen. However, there are also some associated disadvantages, for example: the exoskeleton is molted as the crayfish grows, necessitating recolonization of the new exoskeleton by the ciliate epibiont (Mayén-Estrada and Aladro-Lubel 2000; Fernandez-Leborans and Gabilondo 2006). The advantages for the basibiont Procambarus (Austrocambarus) sp. include protection against desiccation and harmful ultraviolet radiation (Wahl 2008), while disadvantages include the alteration of the body surface and reduced efficiency of locomotion and defense (Fernandez-Leborans 2010).
